# Antimicrobial potential of myricetin-coated zinc oxide nanocomposite against drug-resistant *Clostridium perfringens*

**DOI:** 10.1186/s12866-023-02800-5

**Published:** 2023-03-22

**Authors:** Nada H. Gomaa, Norhan K. Abd El-Aziz, El-sayed Y. El-Naenaeey, Walaa S. Abdelaziz, Alaa H. Sewid

**Affiliations:** 1 Department of Microbiology, Faculty of Veterinary Medicine, Zagazig University, Zagazig, 44511 Sharkia Egypt; 2 Avian and Rabbit Medicine Department, Faculty of Veterinary Medicine, Zagazig University, Zagazig, 44511 Egypt

**Keywords:** *C. perfringens*, Myricetin, Zinc oxide nanoparticles, Real-Time PCR

## Abstract

**Background:**

*Clostridium perfringens* (*C. perfringens*) is an important pathogen in livestock animals and humans causing a wide array of systemic and enteric diseases. The current study was performed to investigate the inhibitory activity of myricetin (MYR), polyvinyl alcohol (PVA), and zinc oxide (ZnO) nanocomposite against growth and α-hemolysin of *C. perfringens* isolated from beef meat and chicken sources.

**Results:**

The overall occurrence of *C. perfringens* was 29.8%. The prevalence of *C. perfringens* was higher in chicken (38.3%) than in beef meat products (10%). The antimicrobial susceptibility testing revealed that *C. perfringens* isolates exhibited high resistance levels for metronidazole (93%), bacitracin (89%), penicillin G (84%), and lincomycin (76%). Of note, 1% of *C. perfringens* isolates were pandrug-resistant (PDR), 4% were extensive drug-resistant (XDR), while 91% were multidrug-resistant. The results of broth microdilution technique revealed that all tested *C. perfringens* isolates were susceptible to MYR-loaded ZnO/PVA with minimum inhibitory concentrations (MICs) ranged from 0.125 to 2 µg/mL. Moreover, the MYR either alone or combined with the nanocomposite had no cytotoxic activities on chicken red blood cells (cRBCs). Transcriptional modifications of MYR, ZnO, ZnO/PVA, and ZnO/PVA/MYR nanocomposite were determined, and the results showed significant down-regulation of α-hemolysin fold change to 0.5, 0.7, 0.6, and 0.28, respectively compared to the untreated bacteria.

**Conclusion:**

This is an in vitro study reporting the antimicrobial potential of MYR-coated ZnO nanocomposite as an effective therapeutic candidate against *C. perfringens.* An in vivo approach is the next step to provide evidence for applying these alternatives in the treatment and prevention of *C. perfringens*-associated diseases.

**Supplementary Information:**

The online version contains supplementary material available at 10.1186/s12866-023-02800-5.

## Background

*Clostridium perfringens* is the most prevalent spore-forming pathogen of humans and livestock animals. It causes numerous diseases with the help of many virulence factors, including its toxins [1], particularly the alpha toxin (CPA), which is produced by all types of *C. perfringens* that targets the plasma membrane leading to hydrolysis of host erythrocytes [2]. Antimicrobial resistance is highly prevalent in *C. perfringens*; many of the isolates carried multiple resistance genes [3], which can be transferred to other bacterial species causing potential threats to animals and human health in the future [4]. Efforts have been made in the research for antivirulence therapy that aims to “disarm” the bacteria by attenuating their virulence determinants, without inhibiting bacterial growth [5]. Therefore, a lesser impact on host commensal microbiota and a lower selective pressure for resistance development are expected when compared to classical antibacterial agents [6]. So, it is highly significant to develop new antibacterial agents with great efficiency against bacteria.

Myricetin (MYR) is a polyphenolic compound, a 3,3′,4′,5,5′,7-hexahydroxyflavone, that derived from vegetables, fruits, nuts, berries, tea, etc. It displays multiple biological effects such as antimicrobial, antioxidant, anticancer, anti-inflammatory, hypoglycemia and cardioprotective activities [7]. The polyvinyl alcohol (PVA) is a hydrophilic and semicrystalline polymer with good biodegradability, biocompatibility, fiber formability, and non-toxicity. It has been greatly used in pharmaceutical and biomedical applications [8]. Recently, nanoparticles (NPs) are increasingly used to target the bacteria as an alternative to antibiotics. One of these NPs is zinc oxide (ZnO), which exhibits marked antibacterial activity even in low amounts, especially against Gram-positive strains [9] by a generation of ions from the surface of NPs that subsequently damage the bacterial cell membrane. Besides, the generation of reactive oxygen species (ROS) induces oxidative stress and cell death [10].

Several shreds of evidence proved that MYR has a potential effect to control bacterial virulence. It possesses an excellent inhibitory effect against α-hemolysin-induced cell damage caused by *Staphylococcus aureus* without inhibiting bacterial growth [11]. This motivated us to explore the possibility of MYR either alone or combined with PVA or ZnO to inhibit the bacterial growth and α-hemolysin activity of *C. perfringens*. If the hypothesis was proved true, the nanocomposite may influence the α-hemolysin-induced cell damage of *C. perfringens*, which is responsible for necrotic enteritis.

## Methods

### Samples

A total of 335 samples were collected from chickens and their products (*n* = 235) and beef meat and their products (*n* = 100) during the period from November 2020 to February 2021. Chicken samples included intestine (*n* = 105), liver (*n* = 50), breast muscle (*n* = 50) and nuggets (*n* = 30), whereas beef meat samples comprised of meat cuts, minced meat, kofta, burger and sausage (*n *= 20 each). Samples of recently slaughtered chickens were obtained from enterprise poultry slaughterhouses, whereas beef meat samples were collected from retail-meat outlets, Abo-Hammad City, Sharkia Governorate, Egypt. They were transported immediately in an ice box to the bacteriology laboratory for further analysis. Euthanasia for birds was performed using a gaseous concentration of 45% carbon dioxide to gently render them unconscious. The animal study was performed regarding the ARRIVE (Animal Research: Reporting In Vivo Experiments) guidelines and approved by the Animal Welfare and Research Ethics Committee, Faculty of Veterinary Medicine, Zagazig University (Approval No. ZU-IACUC/2/F/135/2021).

### Bacteriological analysis and molecular identification

Isolation of *C. perfringens* was performed under anaerobic conditions according to the previously established protocol [12]. The samples were enriched in cooked meat media (Oxoid, Cambridge, UK) at 37 °C for 24 h. The enrichment broth was plated onto Perfringens Agar Base supplemented with egg yolk emulsion and TSC supplement (D-cycloserine 200 mg/vial) (Oxoid, Cambridge, UK). Presumptive *C. perfringen*s colonies were confirmed by the double zone of hemolysis on blood agar, lecithinase activity, motility test, and skim milk coagulation (stormy fermentation) test [13]. The bacterial DNAs were extracted using a QIAamp DNA Mini kit (Qiagen GmbH, Hilden, Germany) according to the manufacturer’s instructions. Polymerase chain reaction (PCR) amplification of the *plc* gene of *C. perfringens* was applied using oligonucleotide primers [14] listed in Additional File 1.

### Antimicrobial susceptibility testing

*Clostridium perfringens* isolates were subjected to antimicrobial susceptibility testing according to the standard procedure for disc diffusion method recommended by the Clinical and Laboratory Standards Institute [15] with a selected panel of 19 standard antimicrobial discs (Oxoid, Cambridge, UK) within 17 different antimicrobial categories. Tested antimicrobials included penicillins [penicillin G (P; 1 unit)], penicillin combinations [amoxycillin-clavulanic acid (AMC; 20/10 µg)], cephalosporines [cefoxitin (FOX; 30 µg)], carbapenemes [imipenem (IPM; 10 µg)], aminoglycosides [gentamycin (CN; 10 µg)], macrolides [erythromycin (E; 15 µg)], quinolones [nalidixic acid (NA; 30 µg)], fluoroquinolones [enrofloxacin (ENR; 5 µg)], sulfonamides [sulfamethoxazole-trimethoprim (SXT; 23.75/1.25 μg)], amphenicols [chloramphenicol (C; 30 µg)], polypeptides [bacitracin (B; 10 µg)], oxazolidones [linezolid (LNZ; 30 µg)], lincosamides [clindamycin (DA; 2 µg) and lincomycin (L2; 15 μg)], tetracyclines [tetracycline (TE; 30 µg)], glycopeptides [vancomycin (VA; 30 µg) and teicoplanin (Tec; 30 µg)], nitroimidazole [metronidazole (MET; 5 µg)], and antimycobacterials [rifampin (RA; 5 µg)]. The interpretive criteria of antimicrobial resistance were determined based on previous reports [16–18] for most antimicrobials, and the British Society for Antimicrobial Chemotherapy (BSAC) for penicillin G, imipenem, clindamycin, and metronidazole [18].

The multiple antimicrobial resistance indices were calculated as previously reported [19]. Pan drug resistance (PDR; resistance to all antimicrobial agents), extensive drug resistance (XDR; resistance to all classes of antimicrobial agents except two or fewer), and multidrug resistance (MDR; resistance to three or more classes of antimicrobial agents) were determined as reported elsewhere [20]. *C. perfringens* ATCC 13,124 strain was used as quality control.

### Preparation of bare ZnO NPs by sol–gel method

Bare ZnO NPs were prepared according to Khan et al., [21] with some modifications**.** At first, 20 g ZnSO_4_ was mixed into 200 mL distilled water and stirred for 30 min at 35–37 °C to produce a zinc sulfate solution. Again, 8 g NaOH powder was weighed, mixed into 80 mL double distilled water (ddH_2_O) and stirred for 20 min at 35 °C for producing 2.5 M NaOH solution. The titration reaction was performed by the addition of 80 mL NaOH into the drop-wise manner accompanied by vigorous stirring. The stirring was continued for 90 min to complete the reaction for obtaining a gel-like product (pH of the final solution is 12). The mixture was dried at 80 °C overnight and calcined in an oven at 250 °C for 4 h; thus, the ZnO NPs were prepared.

### Synthesis of ZnO/PVA/MYR nanocomposite

Twenty mL of PVA polymer (2 g/20 mL ddH_2_O and stirred for 20 min at 50 °C) was added to bare ZnO NPs at a ratio of 1:2, respectively. One gram of MYR was dissolved in 10 mL ddH2O, then the previous solution was added and stirred at 300 rpm for 1 h. The mixture was centrifuged at 10,000 rpm for 10 min to remove the large particles and the unsolved MYR. Finally, ZnO/PVA/MYR nanocomposite was dried at 80 °C overnight and calcined in an oven at 250 °C for 4 h until use.

### Characterization of nanoparticles

**Fig. 1:**
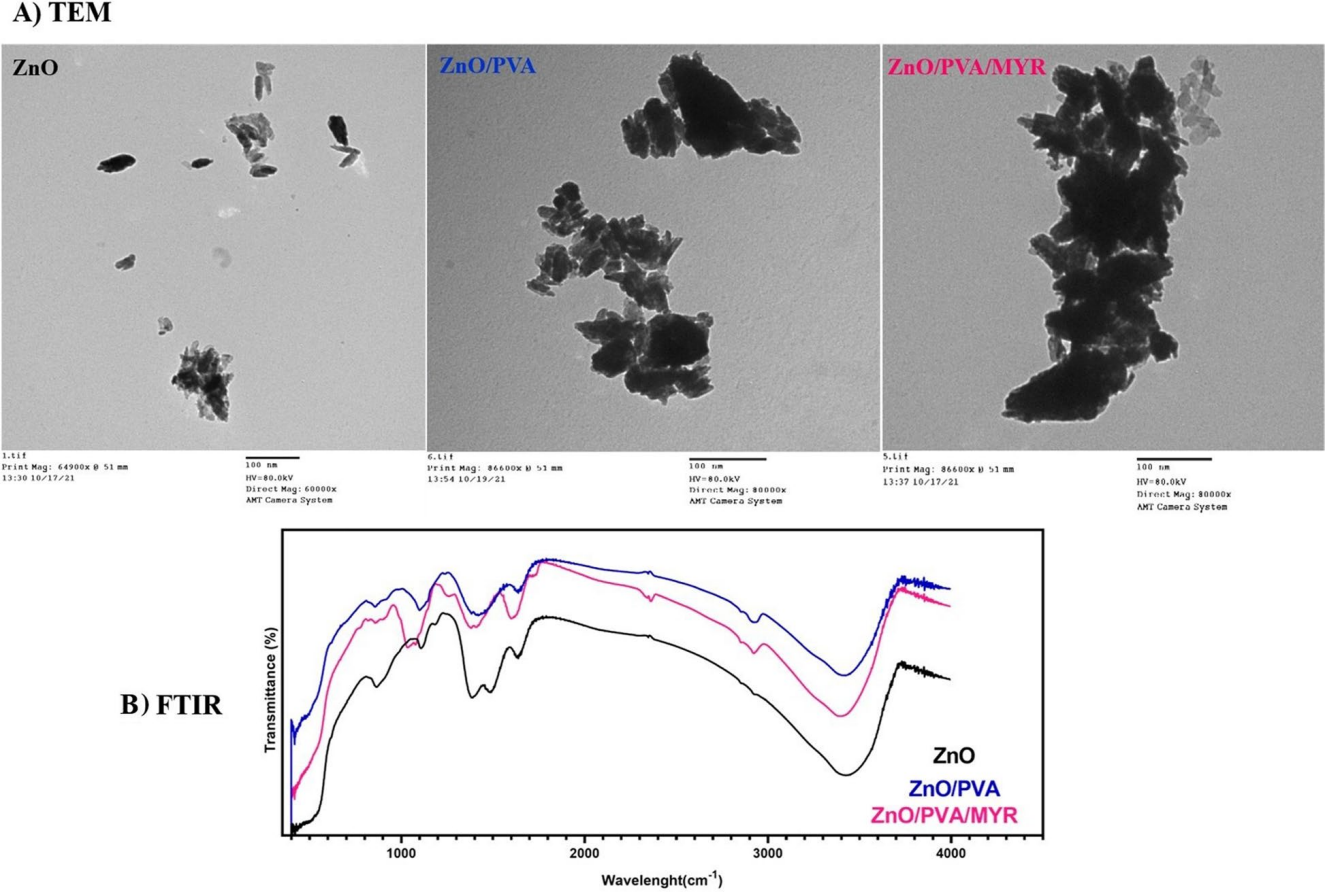
Characterization of MYR-loaded ZnO NPs and PVA polymer. **A**) The morphological surface characterization of prepared NPs by using TEM analysis (ZnO, ZnO/PVA, and MYR/ZnO/PVA); (scale bar = 100 nm). **B**) Fourier-transform infrared spectroscopy (FT-IR) analysis, with characteristic absorption peaks (ZnO, ZnO/PVA, and MYR/ZnO/PVA)

ZnO NPs with particle size lower than 100 nm, PVA with 87–89% hydrolysis degree and molecular weight of 85–124 (Kg/mol), and MYR were purchased from Sigma-Aldrich (St. Louis, MO, USA). Ultra-pure water was used in all preparations. ZnO nanoparticles were coated with PVA as previously described reports [22, 23]. The purchased MYR was prepared as a solution having a concentration of 5 mg /mL and loaded by incubation with 5 mg of ZnO for 12 h to obtain 1:1 loading efficiency as previously described [22]. Transmission electron microscopy (TEM; FE-TEM, Hi-tech model s-3400n, Malaysia) **(** **Fig.** **1****A** **)**, and Fourier transform infrared spectroscopy (FTIR; FTIR-Jascov-650 Spectrophotometer, Thermo Fisher Scientific, USA) **(** **Fig.** **1****B** **)** were carried out for morphological analysis and chemical compound characterization at National Center for Radiation Research and Technology (NCRRT), Atomic Energy Authority, Egypt. A homogenous mixture of ZnO/PVA/MYR nanocomposite was prepared using a magnetic stirrer and a hot plate for 30 min with hot water at 70 °C with continuous stirring until it was completely dispersed.

### Antibacterial activities of nanoparticles

*Clostridium perfringens* isolates exhibited variable antimicrobial resistance profiles (PDR, XDR, and MDR) and showed resistance to the drug of choice, enrofloxacin, that recommended for use only when all other options have failed [24] were examined against the antibacterial effects of MYR, ZnO, ZnO/PVA, and ZnO/PVA/MYR nanocomposite.

Agar well diffusion assay was performed following the previously described procedure [25]. *C. perfringens* bacterial cultures were grown, and bacterial suspensions in sterile saline corresponding to an optical density of 0.5 McFarland (1.5 × 10^8^ CFU/mL) were uniformly spread on Mueller Hinton agar (MHA; Oxoid Ltd., England) using sterile cotton swabs. Wells of 8 mm diameter were cut into each inoculated plate and 100 µL of each tested agent at concentrations of 100%, 50%, and 10% (w/v) were pipetted into each well. Sterile distilled water was used as a negative control, while imipenem acted as a positive control for the bactericide action. The agar plates were incubated under anaerobic conditions for 24 h at 37 °C. The antimicrobial activity was evaluated by measuring the inhibition zone diameter (mm), and the isolates with inhibition zone diameters ≥ 8 mm were considered susceptible. The experiments were carried out in triplicate.

The MICs of the studied antibacterial agents were determined according to the CLSI guidelines in 96-well microtiter plates [15]. 100 µL of various concentrations (1024 to 0.125 µg/mL) of each antibacterial solution in Müeller Hinton broth (MHB; Oxoid Ltd., England), and 100 µL of bacterial suspension previously adjusted to 5 × 10^5^ CFU/mL were pipetted to the wells. Sterile and inoculated MHB without MYR or NPs served as negative and positive controls, respectively. The microtiter plate was incubated under anaerobic conditions for 24 h at 37 °C. The MIC value was considered the lowest concentration of a particular antibacterial agent that could inhibit the bacterial growth.

To determine the minimum bactericidal concentration (MBC), 10 μL of the bacterial suspension starting from the MIC onwards (equal to or two concentrations higher than the MIC) were dropped on MHA and incubated as described above. The lowest concentration of each antimicrobial that killed 99.9% of bacterial growth was considered the MBC [26]. The tolerance levels were determined using the following formula: MBC/MIC [27], and the agent is considered bactericidal when MBC/MIC ratio ≤ 4 [28]. The sub-inhibitory concentration was determined as the highest concentration of antimicrobial agent that showed no effect on survivability and no growth inhibition [29].

### Time-kill kinetics assay

For time-kill curve analysis [30], 1 × 10^5^ CFU/mL for each of of three *C. perfringens* isolates categorized as PDR, XDR, and MDR was incubated in Tryptic Soy Broth (TSB; Oxoid Ltd., England) containing the MIC levels of MYR (1024 µg/mL), ZnO (4–8 µg/mL), ZnO/PVA (2–8 µg/mL), and ZnO/PVA/MYR (0.125–1 µg/mL) nanocomposite.. At determined time points (0, 1, 2, 4, 6, 8, 10, 12, and 24 h), aliquots were aseptically transferred to Tryptic Soy Agar (TSA; Oxoid, England), and the CFU counts were recorded after incubation at 37 °C for 24 h. The experiment was applied in triplicate.

### Cytotoxic effect of MYR and the nanocomposite on chicken red blood cells

Compounds biosafety was evaluated using the hemolysis assay as described previously [31] through reporting the chicken RBCs (cRBCs) lysis, and the amount of hemoglobin released. Blood from three-week-old broiler chickens was collected in EDTA-containing tubes, then the blood was centrifuged and washed four times with 1X phosphate buffer saline (PBS; Oxoid, Cambridge, UK) and reconstituted in 1X PBS to 4% dilution. 100 μL of cRBCs suspension was added to an equal volume of the MIC value of MYR, ZnO, ZnO/PVA, and ZnO/PVA/MYR separately in sterile Eppendorf tubes. PBS (negative control for baseline values) and 1% Triton X-100 (positive control for 100% hemolysis) were used. The suspensions were incubated with continuous agitation (100 rpm) at 37 °C for 3 h on a shaker incubator then centrifuged at 750 rpm for 5 min. 100 μL of the supernatant was added to a 96-well plate, and the absorbance values were determined at 543 nm using a microplate reader (Thermo Fisher Scientific, USA). The assay was calculated as a percentage of cRBCs hemolysis using the following formula: ([Absorbance (treatment − negative control)] / [Absorbance (positive control − negative control]) × 100. The MYR, ZnO, ZnO/PVA, and ZnO/PVA/MYR concentrations at which 50% hemolysis (HC50) occurred were determined and analyzed using GraphPad Prism 8 (GraphPad Software Inc., CA, United States [32].

### Hemolysis inhibition assay

The effect of MYR, ZnO, ZnO/PVA, and ZnO/PVA/MYR nanocomposite on α-hemolysin produced by *C. perfringens* was determined following the protocol described previously [11]. *C. perfringens* isolates were grown overnight in Brain Heart Infusion broth (BHI; Oxoid, Cambridge, UK) at 37 °C under anaerobic conditions then diluted 1:50 in fresh BHI broth with or without MYR, ZnO, ZnO/PVA, and ZnO/PVA/MYR nanoparticles (4X MIC, 2X MIC, MIC, MIC/2, and MIC/4) and incubated anaerobically for 2.5 h to initiate the log-phase cultures. Bacterial supernatants were added to 4% cRBCs suspension and incubated at 37 °C for 3 h then the supernatants were collected by centrifugation, and optical densities were measured at 543 nm. The percentage of hemolysis was determined as described before. The MYR, ZnO, ZnO/PVA, and ZnO/PVA/MYR concentrations at which 50% hemolysis inhibition (IC50) occurred were determined and analyzed using GraphPad Prism 8 (GraphPad Software Inc., CA, United States) [32].

### Transcriptional modulatory effect of MYR alone or combined with nanoparticles on *C. perfringens plc* gene

A reverse transcriptase quantitative PCR (RT-qPCR) was carried out to study the modulatory activity of sub-inhibitory concentrations (MIC/2) of MYR, ZnO, ZnO/PVA, and ZnO/PVA/MYR nanocomposite on the expression of *C. perfringens plc* (α toxin) gene as described elsewhere [33]. The log phase of *C. perfringens* cultures grew in the presence of MYR, ZnO, ZnO/PVA, and ZnO/PVA/MYR or without treatment as a reference control. The cell viability was checked by culture, and the remaining cells were harvested by centrifugation. The cell pellet was mixed with 200 μL RNAprotect Bacteria Reagent (Qiagen, Hilden, Germany). Total RNA was extracted using the RNeasy Mini Kit (Qiagen, Hilden, Germany) according to the manufacturer’s protocol. The residual DNA was removed by DNase digestion column following RNase-Free DNase Set (Qiagen, Hilden, Germany) manufacturer’s protocol. The RNA purity and concentration were determined using Nanodrop measurement. Real-time PCR amplification reaction was prepared in a final volume of 25 µL containing 15 µL of the QuantiTect SYBR Green PCR Master Mix (Qiagen, Hilden, Germany), 1 µL of RevertAid Reverse Transcriptase (Thermo Fisher Scientific, Waltham, Massachusetts, USA), 0.5 µL of each primer of 20 pmoL concentration, 5 µL of RNase-DNase-free water, and 3 µL of RNA template. Quantitative real-time PCR (qPCR) was carried out using primers for the *plc* encoding gene [14] listed in Additional File 1. The amplification condition was performed using an RT-PCR machine (Stratagene, La Jolla, CA, USA). Results were calculated using the comparative cycle threshold method (^ΔΔ^CT method) [34] and normalized against the endogenous control *16S rRNA* [35].

### Statistical analysis

Data were edited in Microsoft Excel (Microsoft Corporation, Redmond, WA, USA). Fisher exact test was performed to detect the significant relationship between different sources and the occurrence of *C. perfringens* as well as antimicrobial resistance. Kruskal—Wallis Test was used to compare the antimicrobial susceptibility testing of MYR, ZnO, ZnO/PVA, and ZnO/PVA/MYR against drug-resistant *C. perfringens* isolates. Bacterial count for the time kill curve analysis was expressed by Log_10_ CFU/ mL. The relationship between the viability of PDR, XDR, and MDR bacteria and the incubation time was fitted by regression analysis [36]. Figures were fitted by the Graph-Pad Prism software 5.0 (Graph Pad, USA). Statistical significance was set at a *P*-value less than 0.05.

## Results

### Prevalence of *C. perfringens* in animal samples

As shown in **Table** 1, the overall prevalence rate of *C. perfringens* was 29.85% (100/335). Out of 235 samples of chicken origin, 90 (38.3%) *C. perfringens* isolates were detected with a higher existence in liver (25/50; 50%) and muscle (22/50; 44%), followed by the intestine (42/105; 40%) and chicken nuggets (1/30; 3.33%). However, a lower percentage of *C. perfringens* was reported in beef meat samples (10/100; 10%) comprising minced meat and meat kofta (3/20; 15% each), meat cuts (2/20; 10%) and meat burger and sausage (1/20; 5% each). Statistical analysis using Fisher exact test revealed a significant variation (*P* = 0.0002) in the existence of *C. perfringens* in chickens and their products, whereas a non-significant difference (*P* = 0.7108) was observed among beef meat and meat products. Collectively, there was a significant difference (*P* = 0.0006) in the occurrence of *C. perfringens* in meat and chicken sources*.*

**Table 1:** Prevalence of *C. perfringens* isolated from animal and human sources

Source (No.)	Sample Type (No.)	Overall prevalence of *C. perfringens* No. (%)	*p*- value
Chickens and chicken products (235)	Breast muscle (50)	22 (44.00)	0.0002
	Liver (50)	25 (50.00)	
	Intestine (105)	42 (40.00)	
	Chicken nuggets (30)	1 (3.33)	
Beef meat and meat products (105)	Minced meat (20)	3 (15.00)	0.7108
	Meat kofta (20)	3 (15.00)	
	Meat cuts (20)	2 (10.00)	
	Meat burger (20)	1 (5.00)	
	Meat sausage (20)	1 (5.00)	
Total	335	100 (29.85)	0.0006

Statistical analysis was performed by Fisher exact test. *P* values ˂ 0.05 represent significant difference

### Antimicrobial resistance of *C. perfringens* isolates

The in vitro antimicrobial susceptibilities of 100 *C. perfringens* isolates against 19 antimicrobial agents are summarized in **Table** 2. The results revealed that high percentages of isolates were resistant to metronidazole (93%), bacitracin (89%), penicillin G (84%), and lincomycin (76%). Moderate levels of bacterial resistance were reported for teicoplanin (60%), linezolid (55%), clindamycin (50%), amoxycillin-clavulanic acid (45%), erythromycin (44%), rifampin (44%), sulfamethoxazole-trimethoprim (42%), tetracycline (40%), nalidixic acid (37%), vancomycin (31%), chloramphenicol (26%), and enrofloxacin (25%). Meanwhile, low percentages of isolates were resistant to gentamycin (15%), cefoxitin (8%), and imipenem (7%). Statistical analysis using Fisher exact test showed significant differences in resistance of the antimicrobial agents among all chicken sources except chicken nuggets (*P* = 0.3918). However, non-significant differences were observed in antimicrobial resistance of *C. perfringens* among all beef meat and meat products except kofta (*P* = 0.0415). In all, there was a significant relationship between antimicrobial resistance of C. perfringens isolates recovered from chicken (*P* = 0.0001) and beef meat samples (*P* = 0.0051).

**Table 2:** Antimicrobial resistance of *C. perfringens* isolated from different sources

					**Number of ** ***C. perfringens*** ** Isolates**						
**AMA** **(Conc.)**	**Chickens and chicken products (** ***n*** ** = 90)**				***P- value***	**Beef meat and meat products (n =10)**					***P- value***
	**Muscle** ** (22)**	**Liver** ** (25)**	**Intestine** ** (42)**	**Chicken nuggets ** **(1)**		**Meat cuts (2)**	**Kofta** ** (3)**	**Burger** ** (1)**	**Sausage (1)**	**Minced meat (3)**	
P (1 U)	18 (81.81)	23 (92.00)	34 (80.95)	1 (100.00)	0.5663	2 (100.00)	2 (66.67)	1 (100.00)	1 (100.00)	2 (66.67)	0.7968
AMC (20/10 µg)	13 (59.09)	11 (44.00)	20 (47.62)	0 (00.00)	0.5825	0 (00.00)	0 (00.00)	0 (00.00)	0 (00.00)	1 (33.33)	0.6281
Fox (30 µg)	2 (9.09)	0 (00.00)	4 (9.52)	0 (00.00)	0.3475	1 (50.00)	0 (00.00)	0 (100.00)	0 (00.00)	1 (33.33)	0.8000
IPM (10 µg)	2 (9.09)	2 (8.00)	3 (7.14)	0 (00.00)	0.9834	0 (00.00)	0 (00.00)	0 (00.00)	0 (00.00)	0 (00.00)	ND
CN (10 µg)	4 (18.18)	4 (16.00)	4 (9.52)	0 (00.00)	0.5855	0 (00.00)	1 (33.33)	0 (00.00)	0 (00.00)	2 (66.67)	0.4553
E (15 µg)	9 (40.91)	8 (32.00)	20 (47.62)	1 (100.00)	0.3716	1 (50.00)	1 (33.33)	1 (100.00)	0 (00.00)	3 (100.00)	0.3571
NA (30 µg)	5 (22.73)	8 (32.00)	18 (42.86)	0 (00.00)	0.3764	1 (50.00)	3 (100.00)	0 (00.00)	0 (00.00)	2 (66.67)	0.2733
ENR (5 µg)	3 (13.64)	4 (16.00)	14 (33.33)	0 (00.00)	0.2228	0 (00.00)	1 (33.33)	0 (00.00)	1 (100.00)	2 (66.67)	0.6571
SXT (23.75/1.25 μg)	9 (40.91)	10 (20.00)	19 (45.24)	0 (00.00)	0.9357	0 (0.00)	2 (66.67)	0 (00.00)	0 (00.00)	2 (66.67)	0.3492
C (30 µg)	6 (27.27)	5 (20.00)	13 (30.95)	0 (00.00)	0.7615	0 (00.00)	0 (00.00)	0 (00.00)	0 (00.00)	2 (66.67)	0.0667
B (10 µg)	19 (86.36)	21 (84.00)	39 (92.86)	1 (100.00)	0.5315	2 (100.00)	3 (100.00)	0 (00.00)	1 (100.00)	3 (100.00)	0.0431*
LNZ (30 µg)	13 (59.09)	13 (52.00)	23 (54.76)	0 (00.00)	0.8211	1 (50.00)	2 (66.67)	0 (00.00)	0 (00.00)	3 (100.00)	0.3571
DA (2 µg)	8 (36.36)	9 (36.00)	28 (66.67)	0 (00.00)	0.0174	1 (50.00)	1 (33.33)	0 (00.00)	0 (00.00)	3 (100.00)	0.2857
L2 (15 µg)	15 (68.18)	18 (72.00)	33 (78.57)	1 (100.00)	0.7316	2 (100.00)	3 (100.00)	1 (100.00)	0 (00.00)	3 (100.00)	0.0404*
TE (30 µg)	5 (22.73)	11 (44.00)	18 (42.86)	0 (00.00)	0.3050	1 (50.00)	1 (33.33)	0 (00.00)	1 (100.00)	3 (100.00)	0.3571
VA (30 µg)	6 (27.27)	5 (20.00)	15 (35.71)	0 (00.00)	0.5829	1 (50.00)	1 (33.33)	0 (00.00)	0 (00.00)	3 (100.00)	0.2857
Tec (30 µg)	13 (59.09)	16 (64.00)	23 (54.76)	0 (00.00)	0.6507	2 (100.00)	2 (66.67)	0 (00.00)	1 (100.00)	3 (100.00)	0.2119
Met (5 µg)	19 (86.36)	24 (96.00)	39 (92.86)	1 (100.00)	0.5060	2 (100.00)	3 (100.00)	1 (100.00)	1 (100.00)	3 (100.00)	0.2119
RA (5 µg)	9 (40.91)	13 (52.00)	17 (40.48)	0 (00.00)	0.7376	1 (50.00)	2 (66.67)	0 (00.00)	0 (00.00)	2 (66.67)	1.000
***P- value***	0.0001*	0.0001*	0.0001*	0.3918	0.0001*	0.2340	0.0451*	0.3918	0.3918	0.1358	0.0051*

Values represent number of *C. perfringens* (%); AMA, antimicrobial agent; Conc., concentration; P, penicillin G benzyl penicillin; AMC, amoxycillin-clavulanic acid; FOX, cefoxitin; IPM, imipenem; CN, gentamicin; E, erythromycin; NA, nalidixic acid; ENR, enrofloxacin; SXT, sulfamethoxazole-trimethoprim; C, chloramphenicol; B, bacitracin; LNZ, lenzolid; DA, clindamycin; L2, lincomycin; TE, tetracycline; VA, vancomycin; Tec, teicoplanin; MET, metronidazole; RA, rifampin. Statistical analysis was done using Fisher exact test. * *P*-values ˂ 0.05 indicate significant differences

The PDR, XDR, and MDR patterns were reported among the analyzed isolates (**Table** 3). In total, 1% (1/100) of *C. perfringens* isolates exhibited a PDR pattern of being resistant to all tested antimicrobial agents **(**Additional File 2**)**, and 4% (4/100) were XDR. The MDR profile was significantly increased among the tested isolates with a percentage of 91% (91/100). However, only 2% (2/100) of the analyzed isolates showed single drug resistance (SDR) and double drug resistance (DDR) patterns each.

**Table 3:** Occurrence of antimicrobial resistance categories in *C. perfringens* from different sources

**Resistance Category**	**Resistance to Antimicrobial Class (** ***n*** ** = 17)**	**Resistance to AMA (** ***n*** ** = 19)**	**No. of Resistant ** *C. perfringens* **(Source)**
			**(** ***n*** ** = 100)**
SDR (n = 2)	1	1	2 (chicken intestine)
DDR (n = 2)	2	2	1 (chicken liver), 1 (chicken muscle)
MDR (n = 91)	3	3	1 (chicken intestine), 1 (chicken liver), 3 (chicken muscle)
	4	4	5 (chicken intestine), 1 (chicken liver), 1 (meat kofta), 1 (meat burger)
		5	1 (chicken intestine)
	5	5	3 (chicken intestine), 5 (chicken liver), 5 (chicken muscle), 1 (chicken nuggets), 1 (meat cuts)
		6	2 (chicken intestine), 1 (chicken liver)
	6	6	1 (chicken intestine), 3 (chicken liver), 2 (chicken muscle), 1 (meat sausage)
		7	1 (chicken intestine)
	7	7	3 (chicken intestine), 1 (chicken liver), 1 (chicken muscle)
		8	2 (chicken intestine), 2 (chicken liver)
	8	8	2 (chicken liver), 1 (chicken muscle)
		9	2 (chicken muscle)
		10	1 (chicken intestine), 1 (minced meat)
	9	9	1 (chicken liver), 1 (chicken muscle),
		10	4 (chicken intestine)
		11	1 (chicken intestine), 1 (meat kofta)
	10	11	1 (chicken intestine), 1 (chicken muscle)
		12	2 (chicken intestine)
	11	12	1 (chicken intestine), 1 (chicken liver)
		13	1 (chicken liver), 1 (chicken muscle), 1 (meat cuts)
	12	13	1 (chicken intestine), 1 (chicken liver)
		14	1 (chicken muscle)
	13	13	1 (meat kofta)
		14	1 (chicken intestine), 1 (chicken liver)
		15	3 (chicken intestine), 2 (chicken liver), 1 (chicken muscle)
	14	15	1 (chicken intestine)
		16	1 (chicken muscle), 3 (chicken intestine), 1 (minced meat)
XDR (n = 4)	15	17	2 (chicken intestine), 1 (chicken liver), 1 (minced meat)
PDR (n = 1)	17	19	1 (chicken muscle)

SDR, single drug-resistance; DDR, double drug-resistance; MDR, multiple drug-resistance; XDR, extensively drug-resistance; PDR, pan drug-resistance, AMA; antimicrobial agent

### Antibacterial activities of MYR either alone or combined with nanoparticles

Ten field *C. perfringens* isolates categorized as MDR (*n* = 5), XDR (*n* = 4), and PDR (*n* = 1) exhibited resistance to at least 13 antimicrobial agents, representing both sample origins including beef meat (*n* = 3), chicken breast muscle (*n* = 2), chicken liver (*n* = 2), and chicken intestine (*n* = 3) were screened for the antibacterial properties of MYR, ZnO, ZnO/PVA, and ZnO/PVA/MYR nanocomposite. The results of agar well diffusion assay revealed different inhibitory zone diameters while using the studied antibacterial agents (Additional File 3 and **Table** 4**)**. Notable inhibition zones of 5–7 and 15 mm were observed with MYR of 50 and 100% concentrations, respectively. While there was no inhibition zone was observed at a lower MYR concentration (10%) among all tested *C. perfringens* isolates. The inhibitory activity value of ZnO NPs against tested *C. perfringens* isolates ranged from 15–20 mm, even at 10% concentration. Statistical analysis using Kruskal – Wallis Test showed that the efficacy of ZnO NPs/PVA polymer (10% concentration) did not significantly differ from ZnO NPs alone. Meanwhile, the inhibition zone diameters of MYR/ZnO/PVA (25–40 mm) were significantly higher (*P* < 0.05) compared with ZnO NPs, ZnO/PVA (15–20 mm each), and IPM control (10–30 mm). Of note, one IPM resistant and another IPM intermediate susceptible *C. perfringens* isolates showed susceptibility to MYR/ZnO/PVA.

**Table 4:** Antimicrobial susceptibility testing of MYR, ZnO, ZnO/PVA, and ZnO/PVA/MYR against* C. perfringens* isolates

Treatments	Inhibition zone	MIC	MBC
MYR	6.3 ± 0.213^d^	1024 ± 1.00^a^	1024 ± 1.00^a^
ZnO	16.00 ± 0.76^c^	3.6 ± 0.82^c^	7.20 ± 1.63^c^
ZnO/PVA	16.00 ± 0.76^c^	2.9 ± 0.69^c^	5.80 ± 1.37^c^
ZnO/PVA/MYR	30.40 ± 1.39^a^	0.625 ± 0.18^d^	1.25 ± 0.36^d^
IPM	21.50 ± 2.11^b^	13.95 ± 2.32^b^	21.50 ± 4.64^b^
*p*-value	< 0.0001	< 0.0001	< 0.0001

The inhibition zone diameters are measured by mm. Minimum inhibitory concentration (MIC) and minimum bactericidal concentration (MBC) values are detected by μg/mL. Breakpoints for *C. perfringens* susceptibility profile according to British Society for Antimicrobial Chemotherapy (BSAC) 2013 was followed for imipenem (IPM); ZnO, zinc oxoid; PVA, polyvinyl alcohol; MYR, myricetin

As presented in **Table** 4, and additional File 3, the MYR had no inhibitory effect on the growth of all tested *C. perfringens* isolates, showing high MIC values (≥ 1024 µg/mL). The MIC values of ZnO NPs and ZnO combined with PVA polymer were 1- 8 µg/mL, while the MBCs were noted at 2- 16 µg/mL with a tolerance level of 2, indicating the bactericidal effect of ZnO NPs. The MIC results illustrated that all tested *C. perfringens* isolates were more vulnerable to MYR-loaded ZnO/PVA than other compounds, with lower concentration values for inhibiting bacterial growth ranging from 0.125 to 2 µg/mL. The lower MIC values of MYR-loaded ZnO/PVA indicate greater antibacterial effectiveness. Meanwhile, IPM exhibited high MIC values of 16–64 µg/mL against three tested *C. perfringens* with no bactericidal activity. Statistically, testing the MYR, ZnO, ZnO/PVA, and ZnO/PVA/MYR for their antimicrobial susceptibilities against drug-resistant *C. perfringens* using Kruskal—Wallis Test showed significant effects through the inhibition zone diameters (*P* = 0.0001), MICs (*P* = 0.0004), and MBCs (*P* = 0.0004) values. Non-significant differences were observed between ZnO and ZnO/PVA for the inhibition zones, MICs, and MBCs, while they differed significantly with ZnO/PVA/MYR and IPM. Also, there were significant differences between all tested antimicrobials in favor of ZnO/PVA/MYR for the inhibition zone diameters and IPM for both MIC and MBC values.

### Time-kill curve analysis

**Fig. 2:**
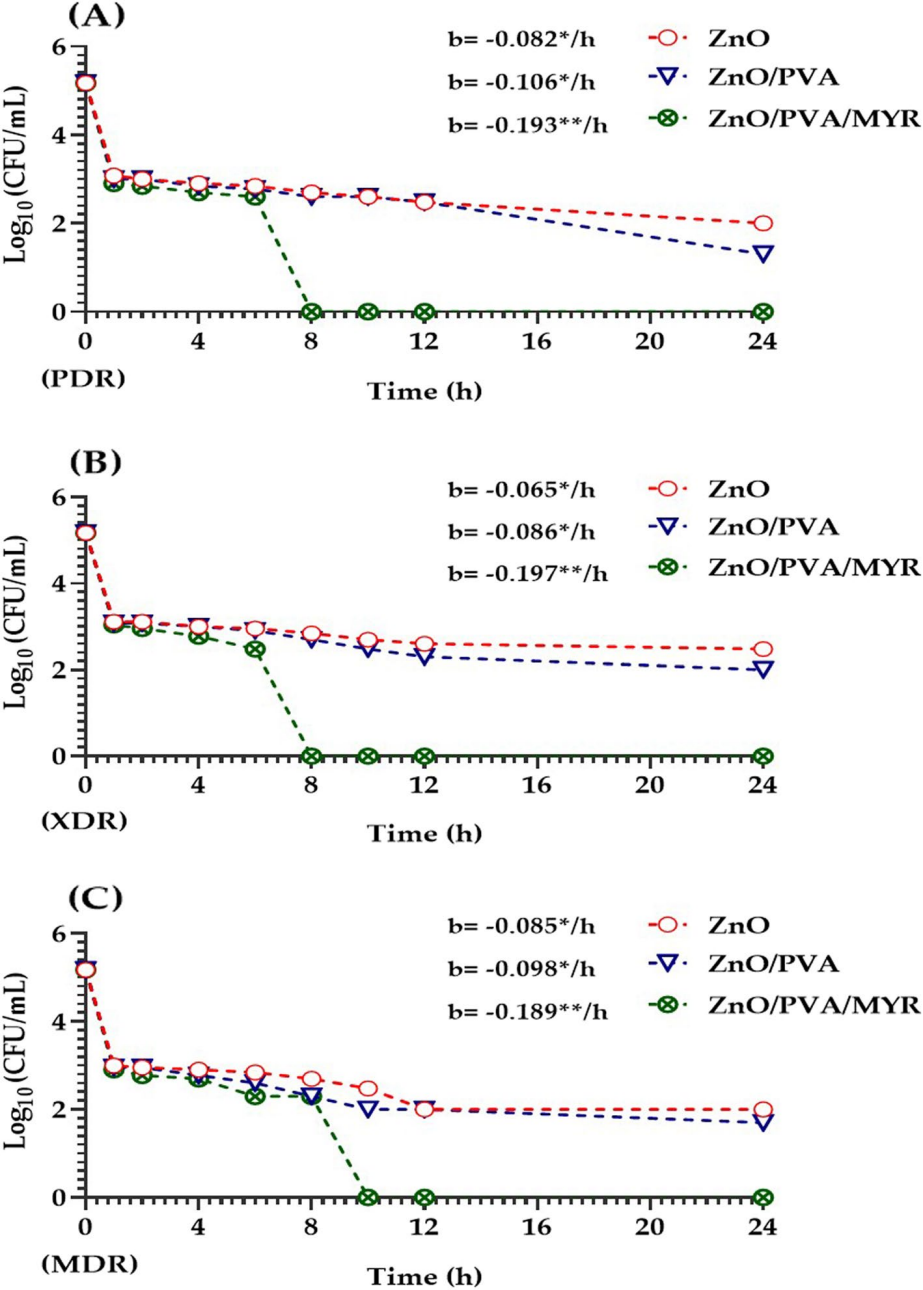
Killing kinetics of the MIC levels of ZnO, ZnO/PVA, and ZnO/PVA/MYR on the viability of three *C. perfringens* isolates categorized as PDR (**A**), XDR (**B**), and MDR (**C**) using the time-kill curve analysis. The bacterial survival was reported at 0, 1, 2, 4, 6, 8, 10, 12, and 24 h incubation time points by the colony forming unit (CFU) assay. PDR, pan drug resistant; XDR, extensive drug resistant; MDR, multi drug resistant. The experiment was applied in triplicate and the regression analysis was performed as previously described method [36]

To assess the killing kinetics of the MIC levels of ZnO, ZnO/PVA, and ZnO/PVA/MYR nanocomposite against three *C. perfringens* isolates (PDR, XDR, and MDR; n = 1 each), the viability of the cells was evaluated in a multi-time point assay (Fig. 2). The regression analysis indicated that each hour increase in incubation time showed significant decrease in the viability of the PDR isolate by 0.082, 0.106, and 0.193 Log_10_ CFU/mL, after treatment by ZnO, ZnO/PVA, and ZnO/PVA/MYR, respectively (Fig. 2-A). Concerning the XDR isolate, the aforementioned regression coefficients were 0.065, 0.086, and 0.197 Log_10_ CFU/mL for the previous order of the nanoparticles (Fig. 2-B). Similarly, for the MDR isolate, the regression coefficient maximized after treatment with ZnO/PVA/MYR being 0.189 Log_10_ CFU/mL/ h followed by ZnO/PVA (b = 0.098 Log10 CFU/mL/ h) and ZnO (b = 0.085 Log_10_ CFU/mL/ h; Fig. 2-C). Collectively, in the three *C. perfringens* isolates of various resistance categories, the ZnO/PVA/MYR- treated bacteria coupled by the greatest slope of the trend line (*P* < 0.01) of time kill curve. Interestingly, exposure of the *C. perfringens* isolates to ZnO/PVA/MYR nanocomposite resulted in 100% inhibition of bacterial cells within 10 h.

### Cytotoxic effect of MYR and nanoparticles on chicken red blood cells

**Fig. 3:**
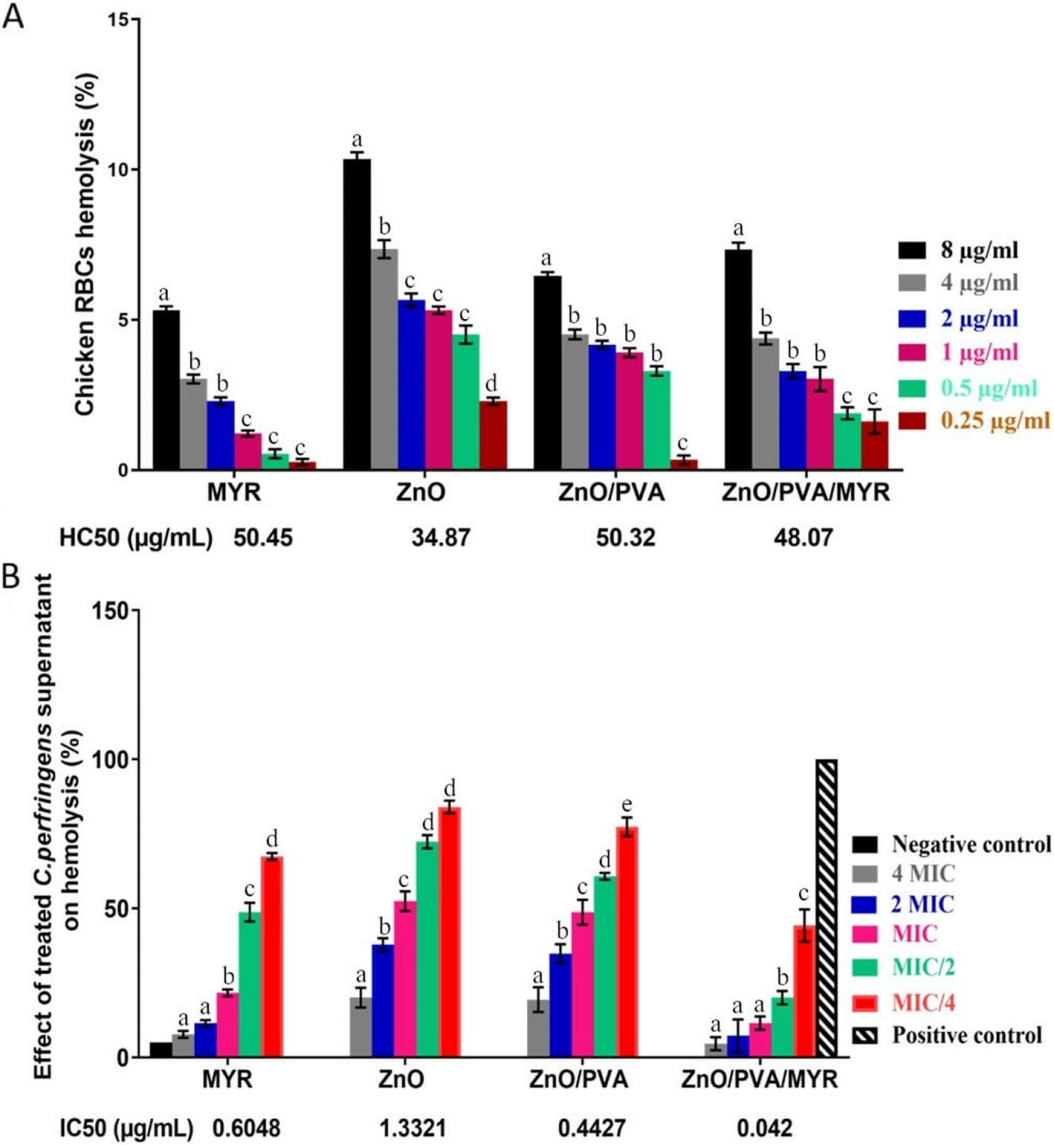
Inhibition of *C. perfringens* culture supernatants (a PDR isolate from chicken breast muscle) hemolysis activity upon incubation with MYR, ZnO, ZnO/PVA, and ZnO/PVA/MYR. **A)** Representative image of bacterial supernatants pre-incubated with varying concentrations (MIC/4, MIC/2, MIC, 2X MIC, 4X MIC) of MYR, ZnO, ZnO/PVA, and ZnO/PVA/MYR compared to untreated positive control and un-inoculated negative control, then the hemolysis activity was determined. **B)** Inhibition of hemolysis activity was performed with varying concentrations and calculated as hemolysis percentage. The half maximal inhibitory concentration (IC50, μg/mL) was represented for each treatment (0.6, 1.3, 0.4, and 0.04). The experiments were done in triplicate and each column represented the mean ± SEM. Different small letters on the same coloured column indicate significant difference (*P* < 0.05)

The possible toxicity and hemolytic activity of MYR, ZnO, ZnO/PVA, and ZnO/PVA/MYR nanocomposite (MICs against tested *C. perfringens* isolates No. 1, 2, and 3 presented in Additional file 3 = 0.25–8 µg/mL) were assessed using chicken erythrocytes. The MYR was exposed to the same concentrations as it had no antibacterial activity against the isolates but its effect was enhanced while loaded on the nanoparticles. Chicken RBCs (cRBCs) incubated with 8 μg/mL of MYR, ZnO, ZnO/PVA, and ZnO/PVA/MYR had 5%, 10%, 6%, and 7% hemolytic activity, respectively (**Fig.** **3****A**) indicating that all the tested compounds did not cause any significant damage or adverse effect to the erythrocytes, unlike the positive control (Triton X-100), did. When cRBCs were incubated with 0.25 μg/mL of MYR, ZnO, ZnO/PVA, and ZnO/PVA/MYR, the percentages of hemolysis reduced to 0.2%, 2%, 0.3%, and 1%, respectively. We also determined the half maximal hemolytic concentrations (HCs50) of MYR, ZnO, ZnO/PVA, and ZnO/PVA/MYR nanocomposite in the presence of various concentrations ranging from 0.25–8 μg/mL (**Fig.** **3****A**). The HCs50 of MYR, ZnO, ZnO/PVA, and ZnO/PVA/MYR for chicken erythrocytes were 50.45, 34.87, 50.32, and 48.07 μg/mL, respectively. It is worth noting that the MIC of MYR-loaded ZnO /PVA against tested *C. perfringens* isolates was ≤ 2 μg/mL. Meanwhile, the HCs50 value was 48.07 μg/mL, which was twenty-four times higher than its MIC, indicating the very low toxicity of MYR-loaded ZnO/PVA, and could be a strong candidate for a new effective therapy.

### Inhibitory effect of MYR, ZnO, and their nanocomposite on C*. perfringens* induced-hemolysis

As shown in **Fig.** **3****B**, the ability of MYR, ZnO, ZnO/PVA, and ZnO/PVA/MYR MICs concentrations (MIC/4, MIC/2, MIC, 2X MIC, and 4X MIC) to hinder the hemolysis induced by a PDR *C. perfringens* culture supernatant (isolated from a chicken breast muscle) was evaluated. The supernatant of each compound-treated bacteria revealed reduced hemolysis of cRBCs in a dose-dependent manner, in which sub-MIC (MIC/4) concentrations of MYR, ZnO, ZnO/PVA, and ZnO/PVA/MYR nanocomposite partially inhibited the hemolytic activity till reach 67.4%, 84%, 77.3%, and 44.2%, respectively; this action is not related to the inhibition of bacterial growth. Notably, this inhibitory effect enhanced with increased concentration; the hemolytic activity of *C. perfringens* culture supernatant on cRBCs significantly decreased (to 4.6%) when cultured with 4X MIC of ZnO/PVA/MYR nanoparticles compared to the control group (100% cRBCs hemolysis). Interestingly, MYR did not affect the growth of *C. perfringens* with the IC50 of 0.6 μg/mL. Furthermore, the IC50 of MYR-loaded ZnO/PVA was 0.042 μg/mL, while the MIC was 1 μg/mL. This potential inhibitory effect with the IC50 value below the MIC value suggests that MYR can reduce the hemolytic activity of *C. perfringens* α-toxin before developing the antibacterial effect. Meanwhile, MYR-loaded ZnO/PVA with a 10-times IC50 (up to 0.42 μg/mL) did not affect the bacterial growth as it was equal to sub-MIC concentration (MIC/2 = 0.5 μg/mL), and it showed an inhibited hemolysis (to 20.02%). The potential inhibitory effects of MIC, MIC/2, and MIC/4 concentrations of MYR, ZnO, ZnO/PVA, and ZnO/PVA/MYR nanoparticles on hemolysis induced by 2 XDR isolates sourced from chicken liver, and intestine are shown in Table 5.

**Table 5:** Inhibition of *C. perfringens* hemolytic activity after incubation with MYR, ZnO, ZnO/PVA, and ZnO/PVA/MYR

No.	Sample code	MYR			ZnO			ZnO/PVA			ZnO/PVA/MYR		
		MIC	MIC/2	MIC/4	MIC	MIC/2	MIC/4	MIC	MIC/2	MIC/4	MIC	MIC/2	MIC/4
1	19M	21.6	48.7	67.4	52.4	72.3	87	48.7	60.7	77.3	11.5	20.02	44.2
2	27L	19.8	35	58	49	69	78	40.3	58.9	69.7	10.8	19.3	40.3
3	125	16.9	29	49	40.2	59	69	31.8	49.3	58.9	9.9	18.7	39.4

The hemolysis activity was calculated as hemolysis percentage

*ZnO* zinc oxoid, *PVA *polyvinyl alcohol, *MYR *myricetin, *MIC *minimum inhibitory concentration, *MBC *minimum bactericidal concentration

### Transcriptional modulation of *C. perfringens* treated with MYR, ZnO, and their nanocomposite

**Fig. 4:**
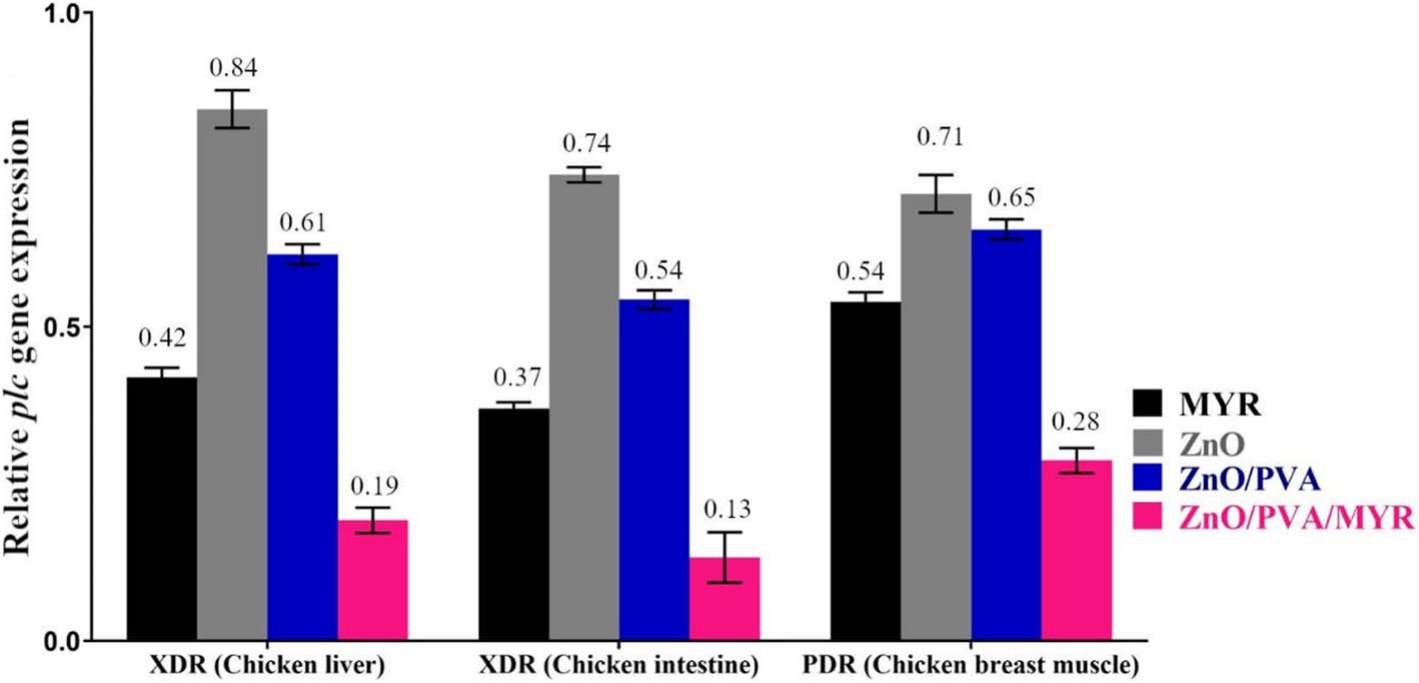
Relative expression of *C. perfringens* alpha-hemolysin (*plc*) upon treatment with sub-MIC (MIC/2) of MYR or ZnO, ZnO/PVA, and ZnO/PVA/MYR. The fold change was determined by qRT-PCR, calculated using the ^ΔΔ^CT method, and normalized comparatively to 16S rDNA expression. The experiments were done in triplicate and each column represented the mean ± SEM

Here, qRT-PCR was used to determine the expression of alpha-hemolysin (*plc* gene) in three *C. perfringens* isolates (one PDR isolated from chicken breast muscle and two XDR isolated from chicken liver and intestine) to investigate the effect of MYR, ZnO, ZnO/PVA, and ZnO/PVA/MYR nanocomposite on the hemolytic activity of *C. perfringens* culture supernatant on cRBCs (**Fig.** **4**). Data analysis indicated that all tested isolates showed down-regulation of the *plc* gene in all treatments when compared to the untreated isolates. The results indicated that the PDR *C. perfringens* isolated from chicken breast muscle treated with the sub-MIC (MIC/2) of MYR, ZnO, ZnO/PVA, and ZnO/PVA/MYR nanocomposite showed significant down-regulation of *plc* gene expression accompanied by reduced fold-change to 0.5, 0.7, 0.6, and 0.28, respectively compared to the untreated bacteria (assigned a value of 1). Thus, the suppression of gene expression level was more vulnerable to MYR-loaded ZnO/PVA than other compounds as it showed decreased fold-changes of 0.28, 0.19, and 0.13 for the PDR and both XDR *C. perfringens* isolates obtained from chicken liver, and intestine, respectively.

## Discussion

*Clostridium perfringens* is an important pathogen causing significant diseases in livestock and humans. A major global challenge is to minimize the use of antimicrobials in the animal industry due to their adverse impacts on public health. Health agencies worldwide are alarmed by the rising concerns of antimicrobial resistance in microorganisms, compromising both human and animal health, and call for the withdrawal of using in-feed antimicrobial growth promoters [37]. Hence, this is the first report demonstrating the in vitro efficacy of the combined antibacterial effect of ZnO nanocomposite coated with the biodegradable PVA polymer and the antivirulence effect of loaded MYR as an attractive target to control antimicrobial resistant *C. perfringens* isolated from different sources.

Herein, the overall prevalence rate of *C. perfringens* was 29.85%, while previous studies reported a lower prevalence rate of 15% in Egypt [38] and 7% in the USA [39].

Necrotic enteritis is an enteric *C. Perfringens*-induced disease of poultry primarily caused by bacterial proliferation in the small intestine and then exotoxins production that can damage the intestinal epithelium [40]. This disease is one of the furthest prevalent illnesses in poultry globally, which causes alarming and significant economic losses of billions per year due to high morbidity and mortality [41]. *C. perfringens* has been implicated in infections of 55.9% of chickens and their products in Egypt [42]. In this study, the prevalence of *C. perfringens* was 38.3% in poultry, which was higher than that reported in a previous study in Egypt (30%) [43] and in Pakistan (25.37%) [44].

Food processing with poor sanitation is a significant source of *C. perfringens* transmission. Here, the prevalence of *C. perfringens* in beef meat and its products was 10%, which was consistent with a previous study on beef meat conducted in china (21.2%) [45], while it was lower than that reported in another study on meat and retail food in Egypt (69.2%) [46]. The difference in the prevalence may be attributed to the different strategic control plans against the disease and the lack of animal and housing strict hygienic measures.

The overuse of antibiotics has led to the emergence of MDR strains, therefore, the first step in preventing the spread of antibiotic resistance is to continue reporting the resistance rates. We provided better insight into different drug resistance patterns (n = 91-pattern) and an alarming increase in PDR, XDR, and MDR profiles while testing 19 antimicrobials among 17 antimicrobial classes. Due to a lack of uniformity in the interpretation of results [47], this is the first study that reported alarming resistance profiles of PDR and XDR *C. perfringens* isolates of 1 and 4%, respectively. Moreover, a worryingly high percentage (91%) of *C. perfringens* isolates showed MDR. Of note, high-dose penicillin is the most common antibiotic therapy used to treat *C. perfringens*-related soft tissue infections in humans, and the possibility of developing resistance to penicillins and β-lactamase is rare. Based on the UNIPROT database, the *bla*_*2*_ gene was initially identified in *Firmicutes*, and *C. perfringens* isolates could acquire this gene from an external source and incorporated it into their genomes [48, 49]. In this study, 84% of *C. perfringens* showed penicillin resistance, which is a worrying percentage compared with a previous study (21.8%) [50].

Due to the emerging XDR and PDR profiles, and the alarming increase of enrofloxacin and imipenem resistance (25 and 7%, respectively), which are recommended for use only when all other options have failed [21], we motivated to search for a new anti-virulence therapy that “disarm” the bacteria by attenuating their virulence determinants.

In this study, we demonstrated the antibacterial efficacy of MYR, ZnO, ZnO/PVA, and ZnO/PVA/MYR against MDR, XDR, and PDR field strains of *C. perfringens* using the in vitro agar well diffusion method, MIC and MBC assays. The results revealed that 100 μL of 10% MYR concentration showed no inhibitory effect on bacterial growth among all tested *C. perfringens* isolates. These findings are supported by a previous study in which the MYR at 100 μM concentration did not exhibit antimicrobial activity against Gram-positive bacteria [11]. However, MYR concentration of 300 μg/disc has an antibacterial effect against *S. aureus* with a 9 mm inhibition zone diameter by the disc diffusion method [51]. Meanwhile, 0.5 mg/mL of MYR concentration showed 16–24 mm inhibition zone diameters against *Enterococcus faecalis*, *Staphylococcus epidermidis*, *Streptococcus pneumonia*, and *Streptococcus pyogenes* [52].

The MIC of MYR against all tested *C. perfringens* field isolates was not detected within the 0.125–1024 μg/mL range. Similarly, other reports revealed that the MICs of MYR against *S. aureus* and *Streptococcus mutans* were > 2000 μg/mL and 512 μg/mL, respectively [52, 53].

In the current study, the inhibitory activity of ZnO NPs tested against *C. perfringens* isolates ranged from 15–20 mm. This result is supported by a previous finding that showed a 24 mm inhibition zone diameter of ZnO NPs against *C. perfringens* [54].

We reported that MYR-loaded ZnO/PVA can remarkably inhibit the bacterial growth with inhibition zone diameters of 25–40 mm and MIC values of 0.125 to 2 µg/mL. Thus, the combination of ZnO NPs with the antivirulence compound MYR can reduce the ZnO dose via coating with the biodegradable PVA polymer without reducing the efficacy against the bacteria, which is considered a reliable drug delivery technology. Moreover, time-kill kinetic study indicated that ZnO/PVA/MYR exhibited bactericidal activity against *C. perfringens* isolates as 100% inhibition of bacterial cells was achieved over the first 10 h of exposure compared to the control untreated isolates.

A previous study revealed the biosafety of MYR as one of the most common plant-derived flavonoid compounds responsible for the flavor and color of fruits and vegetables [55]. All tested concentrations of MYR did not cause any damage to the host erythrocyte membrane [11]. Our data confirmed these previous findings, as the tested MYR, ZnO, ZnO/PVA, and ZnO/PVA/MYR nanocomposite did not cause any significant damage or adverse effect on chicken erythrocytes with the HC50 of all tested compounds ranging from 34.87 and 50.32 μg/mL indicating the very low toxicity of MYR loaded ZnO/PVA, that could be a strong candidate for a new effective therapy.

*Clostridium perfringens* α-toxin is a major virulence factor produced by all *C. perfringens* strains and possessed various biological activities during *C. perfringens* infection. The α-toxin has two enzyme activities, phospholipase C (PLC) and sphingomyelinase (SMase), that induce erythrocytes hemolysis in various animals [56] and cytolysis by directly disrupt the host cell membrane [57]. Also, massive intravascular hemolysis, sometimes leading to anemia occurred in *C. perfringens*-infected patients [58].

Our data presented that MYR and MYR-loaded ZnO/PVA NPs impaired 32% and 55% *C. perfringens* induced hemolysis on chicken RBCs at a concentration of 0.25 μg/mL with IC50 of approximately 0.6 μg/mL and 0.042 μg/mL, respectively, suggesting its ability to reduce the hemolytic activity of *C. perfringens* α-toxin before developing antibacterial stress.

Previous evidence proved that the MYR has a potential effect for controlling bacterial virulence that possesses an excellent inhibitory effect against α-hemolysin-induced cell damage caused by *S. aureus* without inhibiting bacterial growth [11]. Furthermore, MYR is a potential inhibitor that does not exhibit an antimicrobial activity but has been shown to attenuate the hemolytic activity and cytotoxicity of suilysin (SLY) of *Streptococcus suis* [59]. These results led us to investigate the transcriptional effect of MYR and MYR-loaded ZnO/PVA on the *plc* gene expression of *C. perfringens*. The level of expression of the α-toxin gene is involved in the occurrence of necrotic enteritis disease in chickens [60]. The results showed that all tested isolates revealed down-regulation of the *plc* gene in all treatments when compared to the untreated isolates. Meanwhile, the PDR *C. perfringens* treated with 0.6 μg/mL MYR (equal to its IC50 value) and sub-MIC (MIC/2) of ZnO/PVA/MYR NPs modulates alpha-hemolysin transcriptional level to 0.5 and 0.2 fold change, respectively. This is consistent with the previous finding of anti-hemolytic activity of MYR against *S. aureus* due to the direct binding to the Hla toxin, and its capability of inhibiting the production of the α-hemolysin toxin [11].

## Conclusions

The present study highlights the potential of ZnO/PVA nanocomposite combined with MYR as an alternative therapy against MDR, XDR, and PDR *C. perfringens*. This approach exhibits a reliable drug delivery technology without reducing the efficacy against bacteria or adverse effects on chicken erythrocytes. Thus, the massive intravascular hemolysis caused by drug-resistant *C. perfringens*-infection could be mitigated compared to classical less effective antibacterials.

## Supplementary Information

**Additional file 1.**

**Additional file 2.** 

**Additional file 3.**

## Data Availability

The datasets used and/or analyzed during the current study are available from the corresponding author on reasonable request.

## Abbreviations

PVA: Polyvinyl alcohol
MYR: Myricetin
NPs: Nanoparticles
PCR: Polymerase chain reaction
CLSI: Clinical and Laboratory Standards Institute
BSAC: British Society for Antimicrobial Chemotherapy
MAR: Multiple antimicrobial resistance
PDR: Pan drug resistance
XDR: Extensive drug resistance
MDR: Multidrug resistance
TEM: Transmission electron microscopy
FTIR: Fourier transform infrared spectroscopy
NCRRT: National Center for Radiation Research and Technology
MICs: Minimum inhibitory concentrations
MBC: Minimum bactericidal concentration
PBS: Phosphate buffer saline
HC50: 50% Hemolysis
IC50: 50% Hemolysis inhibition
RT-qPCR: Reverse transcriptase quantitative PCR
FDA: Food and Drug Administration
