# Supplementary material for: Antimicrobial potential of myricetin-coated zinc oxide nanocomposite against drug-resistant Clostridium perfringens
Source: BMC Microbiol. 2023 Mar 22;23:79. doi: 10.1186/s12866-023-02800-5 (PMC10031903; doi:10.1186/s12866-023-02800-5)
Supplement: Supplementary file 1 — Additional file 1. [file 12866_2023_2800_MOESM1_ESM.docx]

**Additional Table 1:** Oligonucleotide primers used in this study

| **Gene** | **Oligonucleotide sequence (5′ → 3′)** | **Amplicon size (bp)** | **Reference** |
| --- | --- | --- | --- |
| *plc* gene | F: GTTGATAGCGCAGGACATGTTAA  R: CATGTAGTCATCTGTTCCAGCATC | 402 | (14) |
| *16S rRNA* | F: AAAGATGGCATCATCATTCAAC  R: TACCGTCATTATCTTCCCCAAA | 279 | (35) |

bp, base pair
