# Supplementary figures and images for: Antimicrobial potential of myricetin-coated zinc oxide nanocomposite against drug-resistant Clostridium perfringens
Source: BMC Microbiol. 2023 Mar 22;23:79. doi: 10.1186/s12866-023-02800-5 (PMC10031903; doi:10.1186/s12866-023-02800-5)

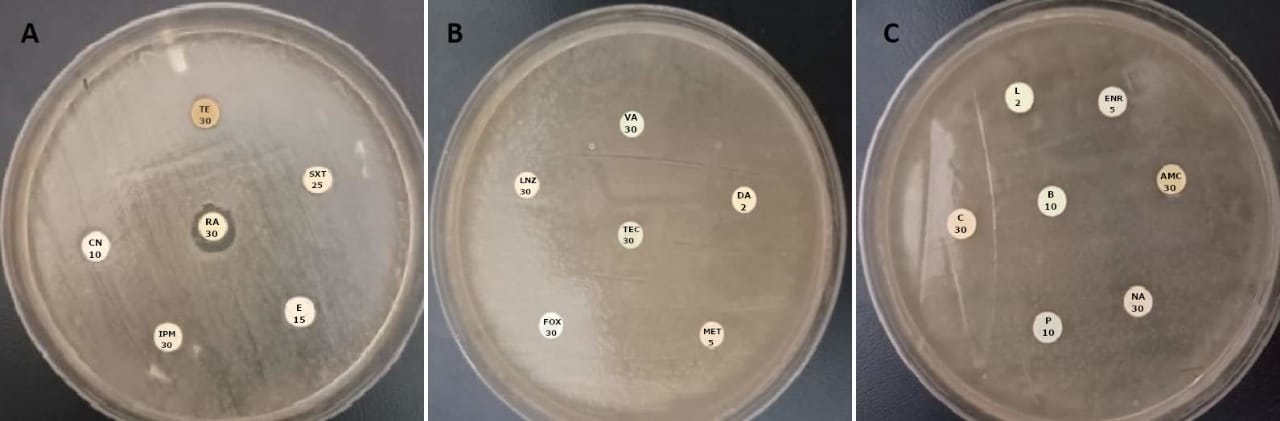

Supplement: Supplementary file 2 — Additional file 2. [file 12866_2023_2800_MOESM2_ESM.jpeg]
