# Supplementary material for: Antimicrobial potential of myricetin-coated zinc oxide nanocomposite against drug-resistant Clostridium perfringens
Source: BMC Microbiol. 2023 Mar 22;23:79. doi: 10.1186/s12866-023-02800-5 (PMC10031903; doi:10.1186/s12866-023-02800-5)
Supplement: Supplementary file 3 — Additional file 3. [file 12866_2023_2800_MOESM3_ESM.docx]

**Additional File 3**: Antimicrobial susceptibility testing of MYR, ZnO, ZnO/PVA, and ZnO/PVA/MYR against 10 *C. perfringens* isolates

| IPM | | | | ZnO/PVA/MYR | | | ZnO/PVA | | | ZnO | | | MYR | | | Sample Code | No. |
| --- | --- | --- | --- | --- | --- | --- | --- | --- | --- | --- | --- | --- | --- | --- | --- | --- | --- |
| Interpretation | MBC | MIC | Inhibition zone | MBC | MIC | Inhibition zone | MBC | MIC | Inhibition zone | MBC | MIC | Inhibition zone | MBC | MIC | Inhibition zone |  |  |
| R | 64 | 64 | 10 | 2 | 1 | 25 | 16 | 8 | 15 | 8 | 4 | 15 | > 1024 | 1024 | 6 | 19M | 1 |
| R | 64 | 32 | 15 | 1 | 0.5 | 30 | 8 | 4 | 20 | 16 | 8 | 20 | > 1024 | 1024 | 7 | 27L | 2 |
| R | 32 | 16 | 15 | 0.5 | 0.25 | 25 | 2 | 1 | 20 | 2 | 1 | 15 | > 1024 | > 1024 | 6 | 125 | 3 |
| I | 8 | 4 | 25 | 0.25 | 0.125 | 35 | 2 | 1 | 15 | 4 | 2 | 15 | > 1024 | 1024 | 6 | 38FM | 4 |
| I | 16 | 8 | 20 | 1 | 0.5 | 29 | 4 | 2 | 15 | 2 | 1 | 15 | > 1024 | 1024 | 6 | 32I | 5 |
| I | 8 | 4 | 25 | 0.5 | 0.25 | 40 | 8 | 4 | 15 | 16 | 8 | 20 | > 1024 | > 1024 | 7 | 220 | 6 |
| I | 16 | 8 | 20 | 2 | 1 | 30 | 4 | 2 | 15 | 4 | 2 | 15 | > 1024 | > 1024 | 5 | 17M | 7 |
| S | 4 | 2 | 25 | 4 | 2 | 30 | 8 | 4 | 15 | 8 | 4 | 15 | > 1024 | 1024 | 6 | 36L | 8 |
| S | 1 | 0.5 | 30 | 0.25 | 0.125 | 30 | 4 | 2 | 15 | 8 | 4 | 15 | > 1024 | 1024 | 7 | 100 | 9 |
| S | 2 | 1 | 30 | 1 | 0.5 | 30 | 2 | 1 | 15 | 4 | 2 | 15 | > 1024 | 1024 | 7 | 214 | 10 |

The inhibition zone diameters are measured by mm. Minimum inhibitory concentration (MIC) and minimum bactericidal concentration (MBC) values are detected by μg/mL.

Breakpoints for *C. perfringens* susceptibility profile according to British Society for Antimicrobial Chemotherapy (BSAC) 2013 was followed for imipenem

R, Resistant; I, Intermediate Susceptible; S, Susceptible; MYR, Myricetin; PVA, Polyvinyl alcohol

The inhibition zone diameters were reported here at a concentration of 50% for MYR and 10% other antibacterial agents under study.
